# Machine Learning-Based Prediction of Mechanical Properties for Large Bearing Housing Castings

**DOI:** 10.3390/ma18174036

**Published:** 2025-08-28

**Authors:** Qing Qin, Xingfu Wang, Shaowu Dai, Yi Zhong, Shizhong Wei

**Affiliations:** 1 School of Mathematics and Statistics, Henan University of Science and Technology, Luoyang 471000, China; qinqing@haust.edu.cn; 2 School of Materials Science and Engineering, Henan University of Science and Technology, Luoyang 471000, China; 13461888763@163.com (X.W.); eastrnk@163.com (S.D.); zylolstve@163.com (Y.Z.); 3 National Joint Engineering Research Center for Abrasion Control and Molding of Metal Materials, Henan University of Science and Technology, Luoyang 471003, China

**Keywords:** large bearing housing castings, machine learning, mechanical property prediction, support vector regression

## Abstract

In modern industrial manufacturing, the mechanical properties of large bearing housing castings are critical to equipment reliability and lifespan. Traditional prediction methods relying on experimental testing and empirical formulas face challenges such as high costs, limited samples, and inadequate generalization capabilities. This study presents a machine learning approach for predicting mechanical properties of ZG270-500 cast steel, integrating multivariate data (chemical composition, process parameters) to establish an efficient predictive model. Utilizing real-world production data from a certain foundry and forging plant, the research implemented preprocessing steps including outlier handling, data balancing, and normalization. A systematic comparison was conducted on the performance of four algorithms: Backpropagation Neural Network (BPNN), Support Vector Regression (SVR), Random Forest (RF), and Extreme Gradient Boosting (XGBoost). The results indicate that under small-sample conditions, the SVR model outperforms other models, achieving a coefficient of determination (R^2^) between 0.85 and 0.95 on the test set for mechanical properties. The root mean square errors (RMSE) for yield strength, tensile strength, elongation, reduction in area, and impact energy are 7.59 MPa, 7.52 MPa, 0.68%, 1.47%, and 5.51 J, respectively. Experimental validation confirmed relative errors between predicted and measured values below 4%. SHAP value analysis elucidated the influence mechanisms of key process parameters (e.g., pouring speed, normalization holding time) and elemental composition on mechanical properties. This research establishes an efficient data-driven approach for large casting performance prediction and provides a theoretical foundation for guiding process optimization, thereby addressing the research gap in performance prediction for large bearing housing castings.

## 1. Introduction

In modern industrial manufacturing, large bearing housing castings serve as critical mechanical components extensively utilized across automotive, aerospace, heavy machinery, and other sectors. Their mechanical properties directly determine equipment operational reliability and service lifespan. ZG270-500 is a commonly used cast steel material exhibiting favorable comprehensive mechanical properties. The tensile strength can reach 583 MPa. The yield strength reaches 332 MPa. In terms of plasticity, the reduction in area is 59% and the elongation is 30.5%. The impact energy can reach 93.8 J. These values indicate that the castings have excellent load-bearing capacity, resistance to deformation and impact, and good quality and applicability. It is widely employed in manufacturing bearing housing castings subjected to moderate loads [1]. However, continuously escalating performance requirements for large bearing housing castings with rough weights exceeding 100 tons challenges conventional casting technologies to meet industrial demands [2,3,4].

Casting technology, as the core process for metal material forming, achieves microstructural optimization of components through precise control of liquid metal solidification processes. Various simulation methods provide critical tools for elucidating performance control mechanisms in casting processes [5]. Traditional methods for predicting mechanical properties of castings primarily rely on experimental testing and empirical formulas. Although these approaches partially fulfill engineering requirements, they exhibit significant limitations. Experimental testing, which demands substantial time and resources while permitting analysis only on limited specimens, making comprehensive evaluation of complex casting processes and diverse influencing factors challenging, includes chemical composition analysis involving the determination of elements such as C and Mn, and mechanical property testing including tensile and impact tests [6]. Empirical formulas are typically derived under specific experimental conditions and simplified assumptions, failing to account for various nonlinear factors and interactions inherent in actual production. Consequently, their prediction accuracy and generalization capability are constrained.

With the rapid advancement of computer technology and data science, machine learning has gained increasingly extensive application in materials science as a powerful tool for data analysis and modeling. Machine learning can automatically extract complex patterns and regularities from vast datasets without relying on explicit physical or mathematical models, making it particularly suitable for addressing the prediction of mechanical properties in castings characterized by high nonlinearity, multivariate interactions, and noise interference [7,8,9,10]. By constructing appropriate machine learning models, various influencing factors in the casting process—including chemical composition, melting parameters, cooling conditions, and heat treatment processes—can be effectively integrated to enable accurate prediction of mechanical properties for ZG270-500 bearing housing castings [11]. Current research applying machine learning methods to predict casting mechanical properties has achieved notable results. Pei et al. [12] developed Lasso and XGBoost models to predict ultimate tensile strength (UTS) and elongation (EL) of extruded ZL101 aluminum alloy castings. Under 25 small-sample conditions, Bayesian optimization achieved UTS prediction with R^2^ = 0.8142 and EL prediction with R^2^ = 0.7573. Shahane et al. [13] employed a multilayer perceptron neural network to replace empirical models, enabling efficient yield strength prediction for Al-Mg alloy castings with errors consistently controlled within 2.04%. Jaśkowiec [14] et al. compared classical machine learning algorithms with neural networks and used input data such as microstructure images of castings to predict mechanical parameters such as tensile strength. The accuracy rate of AdaBoost on imbalanced datasets reached 83.4%. Hong [15] et al. used four machine learning algorithms to predict the thermoplasticity of cast steel based on composition and thermal conditions. The neural network model was the best, and the three RMses of low-temperature limit were 20.6 °C, 15.2 °C, and 27.6 °C, respectively. Wilk-Kołodziejczyk [16] et al. constructed mechanical property prediction models such as UTS and YS for austempered ductile iron (ADI) cast iron using multiple machine learning algorithms. The gradient boosting algorithm performed the best, for instance, the R^2^ of UTS reached 0.8243 and that of YS was 0.8513. In addition, machine learning also has extensive applications in other steel fields [17,18]. While machine learning applications in material performance prediction continue to expand with intelligent manufacturing advancements, current research predominantly focuses on conventional-sized materials, with studies on large-scale castings remaining scarce.

This study focuses on predicting the mechanical properties of large ZG270-500 cast steel bearing housing castings. Production data from a foundry-forging plant were utilized, encompassing chemical composition, process parameters (e.g., pouring speed, normalizing holding time), and mechanical property indices. The dataset was constructed by eliminating outliers and performing data balancing and normalization. Comparing four models—BPNN, SVR, RF, and XGBoost—SVR demonstrated optimal performance with small samples, achieving an R^2^ of 0.89–0.96 on the test set. Validation with new specimens showed relative errors between predicted and measured values below 4%, demonstrating its applicability. SHAP value analysis revealed the influence mechanisms of elements (e.g., P, Cr) and process parameters (e.g., pouring speed, normalizing holding time) on mechanical properties. This provides a basis for process optimization and fills a research gap in performance prediction for large bearing housing castings.

## 2. Method

### 2.1. Data Collection

The dataset used in this study was obtained from a domestic casting and forging plant specializing in the production of large-scale forgings. A total of over 70 valid data records under room temperature conditions were collected, each corresponding to an independent production experiment and containing complete information on chemical composition and process parameters. All data were recorded in real time by the plant’s technical staff during actual production, thus accurately reflecting onsite process conditions and material states. As such, the dataset possesses high authenticity and reliability, providing a solid and trustworthy foundation for the subsequent development and performance analysis of the prediction models.

**Figure 1:**
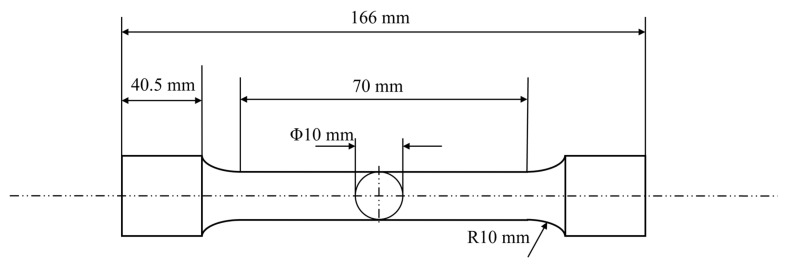
Tensile specimen size information.

For mechanical property testing, standard tensile specimens were machined according to the geometric dimensions shown in Figure 1 and tested using a universal testing machine (SLFL 100 kN, Shimadzu, Kyoto, Japan). The relationship between load and deformation during the tensile process was recorded to plot the stress–strain curve, from which key mechanical properties such as yield strength, tensile strength, and elongation at break were extracted. Impact toughness tests were conducted using a microcomputer-controlled pendulum impact testing machine (PTM 2302-C, SUNS, Shenzhen, China), which precisely measured the impact energy of the material, thereby characterizing its toughness under dynamic loading conditions.

### 2.2. Machine Learning Process

**Figure 2:**
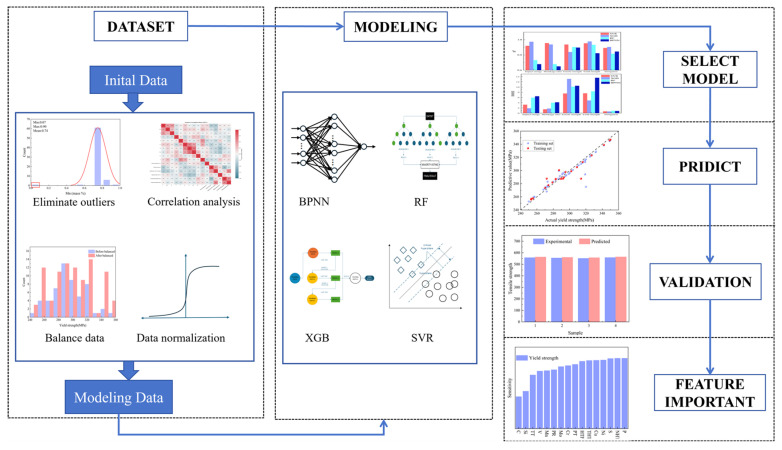
Overall process of machine learning.

This study employs a data preprocessing pipeline to establish a foundation for modeling. Prior to model training, the raw data undergo rigorous preprocessing procedures: (1) outlier detection and elimination using the 3σ criterion; (2) standardization of multi-source heterogeneous feature data through min–max normalization; (3) resolution of class imbalance via oversampling techniques. During the model construction phase, the research team systematically evaluated four representative machine learning algorithms—RF, SVR, BPNN, and XGBoost—using the preprocessed high-quality dataset. Through a comprehensive comparison of two key performance metrics (R^2^ and RMSE), XGBoost was identified as the optimal predictive model. Furthermore, SHAP interpretability analysis not only identified critical process influencing factors but also quantified the contribution of each parameter to mechanical properties. Building upon these findings, this study innovatively developed a closed-loop intelligent regulation framework (Figure 2) encompassing “data acquisition-model prediction-process optimization.” This framework continuously enhances prediction accuracy through real-time data feedback mechanisms, providing a reliable technical pathway for the intelligent upgrading of cast steel production processes.

### 2.3. Data Processing

Data processing plays a critical role in the construction of machine learning models. The success of machine learning models is largely determined by the quality of the dataset. High-quality datasets typically exhibit characteristics such as accuracy, completeness, and regularity, which are essential for machine learning models to achieve strong generalization capability and predictive performance. Therefore, implementing data preprocessing to fully manifest these characteristics constitutes a necessary step for enhancing model prediction accuracy. The data in this study were collected from actual production records at a foundry-forging plant, comprising over 70 entries. Each entry represents an independent production sample.

#### 2.3.1. Outlier Elimination

**Figure 3:**
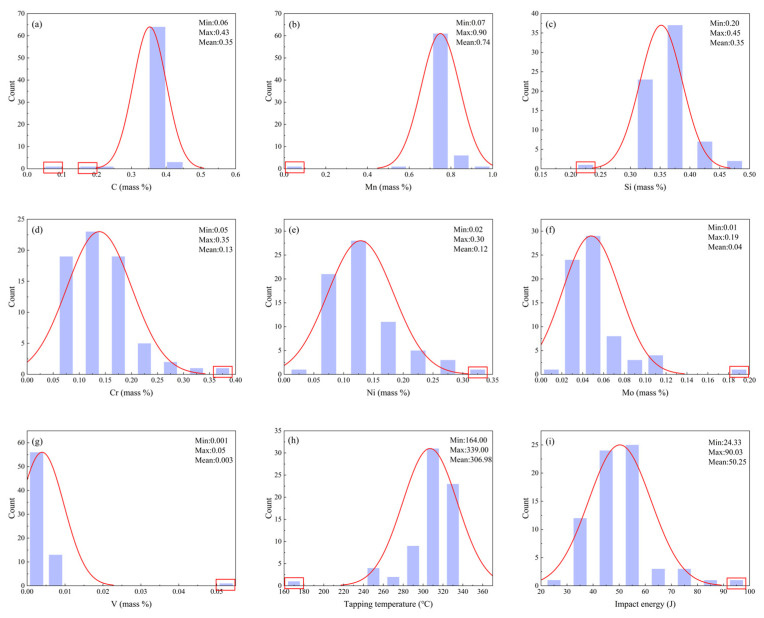
Data distribution before and after eliminating outliers (**a**) C; (**b**) Mn; (**c**) Si; (**d**) Cr; (**e**) Ni; (**f**) Mo; (**g**) V; (**h**) Tapping temperature; (**i**) Impact energy.

Data collected in industrial environments are often subject to outliers due to factors such as special operating conditions, measurement errors, or data entry mistakes. These outliers can lead to systematic biases in the prediction results, manifesting as either an overestimation or underestimation of the predicted values, which significantly reduces the model’s prediction accuracy and fitting ability. To improve data quality, this study employs the 3σ principle based on a normal distribution for outlier detection and removal. Specifically, the 3σ rule classifies samples outside the range of μ ± 3σ as potential outliers, where σ represents the standard deviation of the data, and this range covers approximately 99.73% of the normal data distribution. Data points beyond this range are initially marked as potential outliers. However, considering the complexity and variability of industrial data, this study further incorporates expert judgment and material physics principles to comprehensively analyze and confirm these potential outlier candidates. By leveraging experts’ understanding of the data generation context and adherence to physical laws, this approach effectively distinguishes between true outliers and extreme but plausible data, ensuring the scientific validity and reasonableness of the removal process. Figure 3 illustrates the distribution changes of key parameters before and after outlier removal, the distribution of outliers is shown in the red box. The red line represents the distribution of the parameters, and the overall distribution shows a normal distribution, while Table 1 provides detailed statistical features of the parameters post-outlier removal. This method significantly enhances the validity of the data, providing a solid foundation for subsequent modeling and predictive analysis.

#### 2.3.2. Correlation Analysis

**Figure 4:**
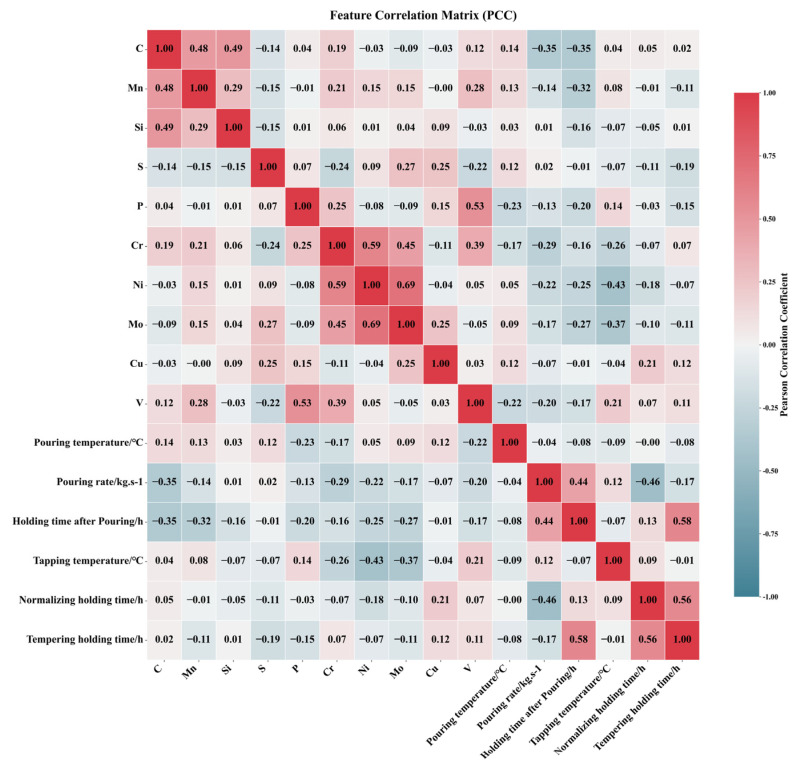
Pearson correlation coefficient graph among various features.

**Figure 5:**
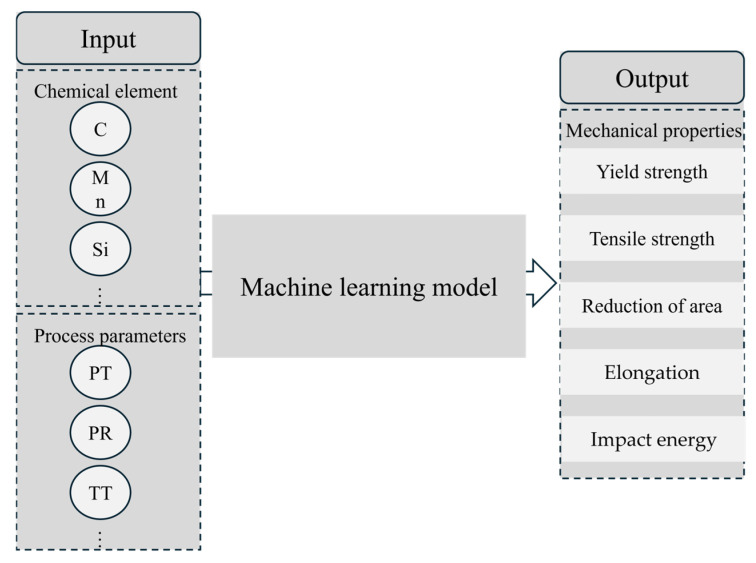
Machine learning input–output diagram.

The Pearson correlation coefficient (PCC) heatmap serves as a powerful and widely adopted visualization tool in exploratory data analysis. It intuitively presents pairwise Pearson correlation coefficients between variables through a color-coded matrix, revealing underlying correlation structures within datasets and guiding subsequent analytical directions. For instance, Dai et al. [19] employed PCC heatmaps to elucidate intrinsic relationships among features affecting the hardness of dispersion-strengthened tungsten alloys. Figure 4 displays the PCC heatmap for inter-feature correlations, utilizing color intensity to represent correlation strength: deep red regions indicate strong positive correlations where an increase in one feature value corresponds to an increase in another, while navy blue regions signify pronounced negative correlations, where an increase in one feature value accompanies a decrease in another. Light-colored areas denote weak correlations (approaching zero) between features, suggesting potential independence. Feature pairs with |PCC| ≥ 0.7 are typically considered highly correlated and thus removed. In Figure 4, the correlation coefficient between Ni and Mo content (0.69) approaches this threshold. Given that tree-based models exhibit insensitivity to multicollinearity, both Random Forest and XGBoost models retain these features in our analysis. In addition, Figure 5 presents the input and output parameters used in the machine learning prediction. The input parameters include composition and process variables, while the output parameters are mechanical properties such as yield strength.

#### 2.3.3. Data Balancing

**Figure 6:**
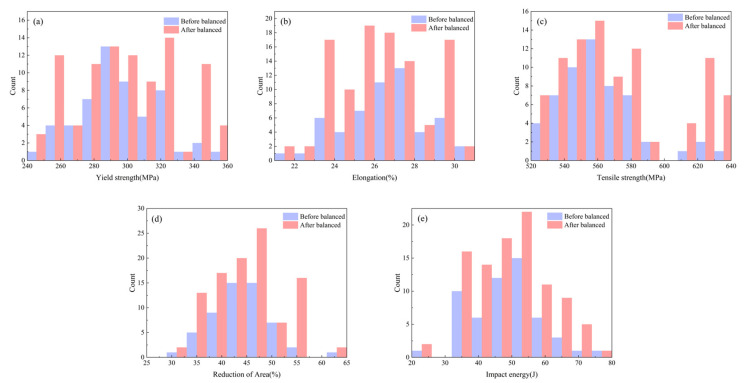
Balanced data distribution (**a**); Yield strength; (**b**) Elongation; (**c**) Tensile strength; (**d**) Reduction in area; (**e**) Impact energy.

Establishing relationships between mechanical properties and process parameters/composition requires sufficient production data for modeling. Practical production constraints often result in discrete and imbalanced collected data. Predictive models built on such data yield inconsistent prediction patterns and reduced accuracy, as information from sparsely distributed regions is inadequately learned during training, compromising local prediction precision. When modeling tensile strength of alloy steels, Zhang et al. [20] addressed data imbalance by clustering similar data into groups and selecting fixed numbers of representative samples per group to mitigate over-concentration. However, this approach risks losing critical data points. Thus, we implement oversampling (data replication) to augment infrequent data and increase their representation. Figure 6 contrasts balanced data distribution.

#### 2.3.4. Data Normalization

Data normalization transforms datasets into a fixed numerical range, typically scaling values to [0, 1] or (−1, 1) decimals. This method facilitates data processing by mapping data to a [0, 1] interval, enhancing computational efficiency. It transforms dimensional expressions into dimensionless quantities, enabling comparison and weighting of metrics across disparate units/scales. As a computational simplification technique, it converts dimensional variables to unitless scalars.

The method achieves proportional scaling of raw data using variable maxima and minima to transform data into a specified bounded range, eliminating dimensional and magnitude effects, thereby resolving metric heterogeneity through variable weight adjustment. Equation (1) projects feature parameters onto the [0, 1] interval [21]:

(1) $$X^{\prime }=\frac{X-X_{min}}{X_{max}-X_{min}}$$

where *X* denotes the raw feature value, $X_{min}$ and $X_{max}$ represent the minimum and maximum values observed in the dataset, respectively, and *X*′ indicates the normalized value within the [0, 1] range.

### 2.4. Model Construction and Selection

The processed dataset was split into training and test sets at an 8:2 ratio, with 10-fold cross-validation applied to the training set for training and evaluating various machine learning models. Model accuracy was assessed using performance metrics such as RMSE, and the best-performing model was subsequently employed to predict the mechanical properties of ZG270-500.

As a fundamental algorithm of artificial neural networks, backpropagation (BP) exhibits robust nonlinear modeling capabilities. The algorithm calculates output layer errors, propagates these errors backward through network layers, and systematically adjusts synaptic weights and thresholds to optimize the learning trajectory, thereby iteratively minimizing prediction errors [22]. Furthermore, its activation functions and multi-layer architecture enable learning highly complex nonlinear relationships and automatically capturing intricate feature interactions, reducing reliance on feature engineering. Models trained with BP possess strong generalization capability, allowing reliable predictive modeling even with small, high-quality datasets. For instance, Liu et al. [23] achieved significant results using BP neural networks for performance prediction on small datasets.

Because SVR is derived from support vector machine theory, SVR identifies an optimal hyperplane in feature space that minimizes prediction deviations across the dataset. Its distinctive ε-insensitive loss function imposes zero penalty for errors within a specified tolerance threshold [24]. This regularization strategy enhances generalization by balancing empirical risk minimization with controlled error tolerance. The algorithm maintains robust performance in high-dimensional, small-sample scenarios, as demonstrated by Wang et al. [25] for large cylindrical component properties.

The Random Forest Regression algorithm ensemble method employs bootstrap aggregation (bagging) to enhance stability. Multiple decorrelated decision trees are trained via random subspace sampling with replacement. Final predictions aggregate individual tree outputs through averaging, intrinsically reducing variance and mitigating overfitting [26]. The framework provides inherent feature importance evaluation through permutation-based analysis.

Regarding XGBoost as an optimized gradient boosting implementation, XGBoost iteratively constructs additive tree models. Second-order Taylor approximations of loss functions improve gradient estimation precision, while integrated regularization terms control model complexity [27]. Block-structured data partitioning enables parallel computation, accelerating training for large-scale datasets.

This study employs R^2^ and RMSE as evaluation metrics for comprehensive model assessment. The coefficient of determination quantifies the model’s goodness-of-fit, while the root mean square error provides an absolute measure of prediction error. Their mathematical formulations are expressed as Equations (2) and (3):

(2) $$R^{2}=1-\frac{\sum_{i}{\left(y_{i}-\hat{y_{i}}\right)}^{2}}{\sum_{i}{\left(\bar{y_{i}}-y_{i}\right)}^{2}}$$

(3) $$RMSE=\sqrt{\frac{1}{m}\sum_{i=1}^{m}{\left(y_{i}-\hat{y_{i}}\right)}^{2}}$$

where$\;y_{i}$ denotes observed values, $\hat{y_{i}}$ represents predicted values, and $\bar{y_{i}}$ signifies the mean of observed values.

## 3. Results and Discussion

### 3.1. Model Performance Comparison

This study systematically evaluated four classical machine learning models for predicting five key mechanical properties of steel. Table 2 and Table 3 present evaluation metrics for training and test sets across algorithms. Comparative analysis reveals that while XGBoost effectively captures interactions between alloy composition and process parameters and achieves excellent training performance (R^2^ ≈ 0.99), it exhibits significant overfitting on the test set—for instance, RMSE for tensile strength (TS) prediction surges from 0.07 to 13.12. Random Forest (RF) demonstrates superior stability (test R^2^ = 0.68–0.93) though with comparatively lower predictive accuracy, which may stem from the piecewise constant prediction nature of its decision tree base-learners. Although BPNN performs well on certain metrics (e.g., elongation E: test R^2^ = 0.84, RMSE = 0.82), its overall performance shows substantial volatility, likely resulting from the conflict between neural networks’ data volume requirements and the small-sample characteristic of the dataset.

**Figure 7:**
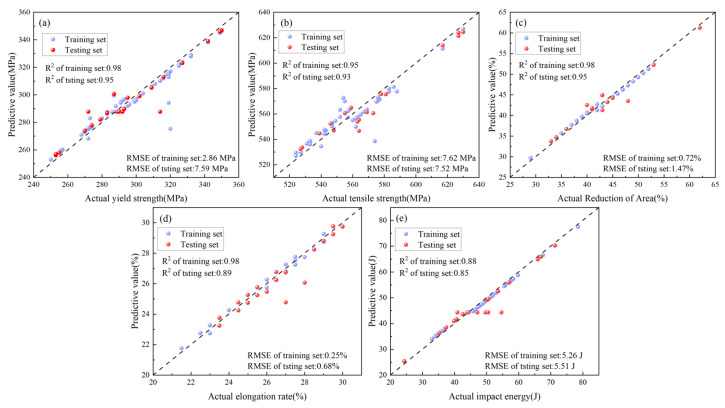
Different performance results predicted by SVR (**a**) Yield strength; (**b**) Tensile strength; (**c**) Reduction in area; (**d**) Elongation; (**e**) Impact energy.

Figure 7 presents SVR prediction results for impact energy, elongation, yield strength, tensile strength, and reduction in area. Results demonstrate that SVR leverages unique advantages in small-sample high-dimensional scenarios (training-set R^2^ = 0.88–0.98, test-set R^2^ = 0.85–0.95), combining the RBF kernel’s capacity for nonlinear relationships and robustness to outliers to achieve optimal comprehensive predictive performance. Notably, SVR excels in elongation prediction (test-set RMSE = 0.68) while exhibiting relatively higher error in yield strength prediction (RMSE = 7.60). This differential performance likely reflects varying complexity in mapping relationships between mechanical properties and input features. These findings not only validate SVR’s distinctive strengths in material property prediction but also delineate applicability boundaries of ML algorithms in materials science, providing critical references for model selection in subsequent research.

### 3.2. Experimental Validation of the Optimal Model

#### 3.2.1. Experimental Method

**Figure 8:**
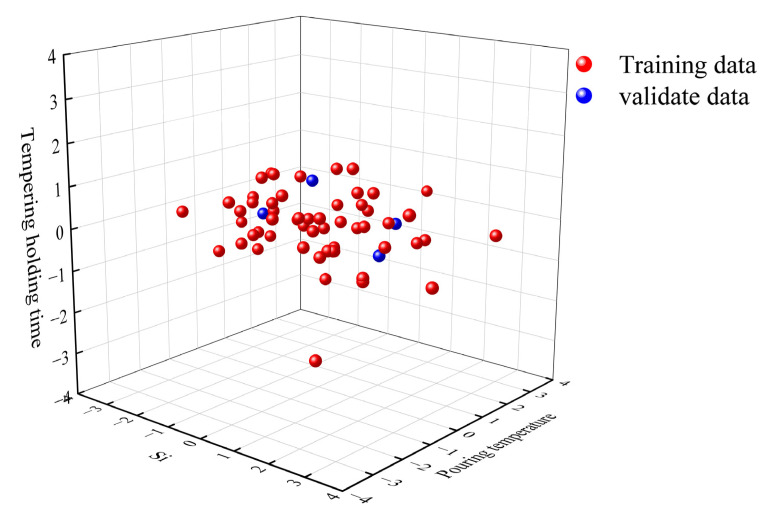
PCA analysis results of the new samples.

To validate the predictive capability of the proposed model for the mechanical properties of ZG270-500 steel, new samples were collected in strict accordance with the data acquisition standards and conditions used for the training dataset, ensuring consistency in sample origin, environmental conditions, and equipment parameters, thereby fundamentally minimizing distributional differences. The newly fabricated ZG270-500 steel specimens were produced following the standard production line process, with their chemical compositions and processing parameters listed in Table 4. To further assess the distributional consistency between the new samples and the training data, principal component analysis (PCA) was employed. Specifically, the principal component space that captures the maximum variance of the data was first computed from the training set, and the new sample dataset was then projected onto this space. Differences between the two datasets were evaluated in terms of projected distribution, reconstruction error, and subspace angles; datasets from the same distribution are expected to exhibit close values for these metrics, whereas significant differences would indicate distributional shifts. Compared with directly computing distributional differences in high-dimensional space, PCA offers advantages such as reduced computational cost after dimensionality reduction, preservation of the primary structural information, ease of visualization and interpretation, and the ability to intuitively display the similarities and differences between datasets in a low-dimensional space. As shown in Figure 8, the new sample data exhibit a high degree of overlap with the training data in the projection space, indicating high prediction accuracy and robustness under these conditions. Furthermore, a comparative analysis between the experimental measurements of the new samples and the model predictions confirms the model’s strong applicability and reliability in industrial settings.

#### 3.2.2. Prediction

**Figure 9:**
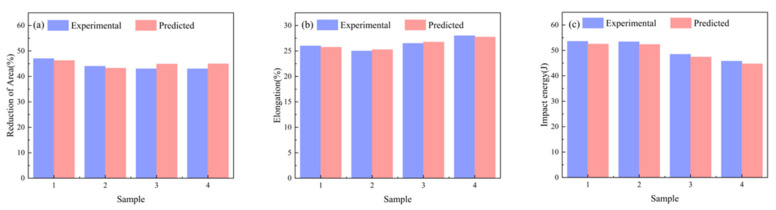
Comparison between predicted and measured values across varying performance (**a**) Reduction in area; (**b**) Elongation; (**c**) Impact energy.

**Figure 10:**
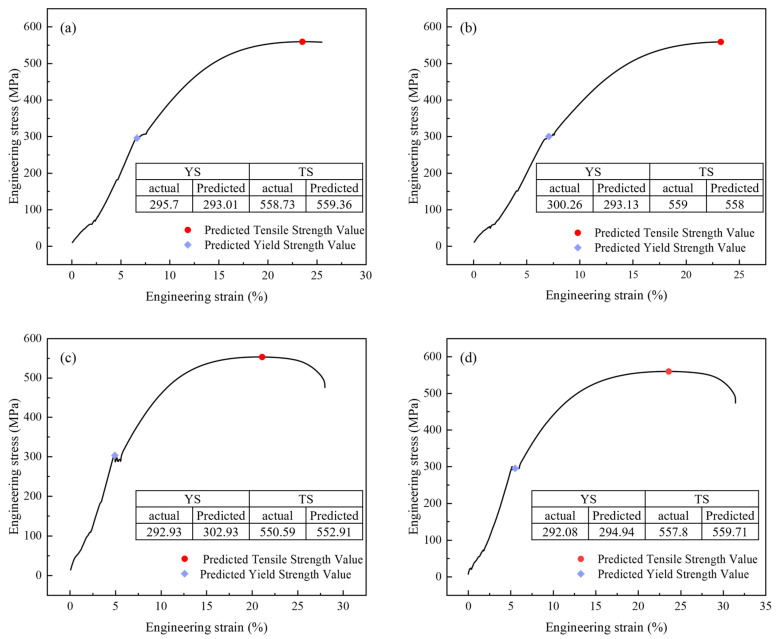
Prediction of the tensile curve of four samples (**a**) sample 1; (**b**) sample 2; (**c**) sample 3; (**d**) sample 4.

Figure 9 compares actual measurements and model predictions of tensile properties for four ZG270-500 steel sample groups. Figure 9a,b, reveal maximum relative errors of 2.12% for reduction in area and 3.84% for impact energy, while Figure 9c indicates a maximum relative error of 2.05% in impact energy measurement. Figure 10 displays engineering stress–strain curves for four test specimens, comparing measured versus predicted yield strength and tensile strength to validate the performance prediction model. The curves represent experimentally measured tensile responses, with colored geometric markers indicating predicted yield and tensile strengths. These curves exhibit characteristic mechanical responses—elastic deformation, plastic flow, and necking. Close alignment between predicted and actual values is observed, with minimal deviations across all predictions, demonstrating the model’s precision in capturing critical mechanical properties. This serves as a reliable basis for performance pre-screening and process optimization in material development.

### 3.3. SHAP Value Analysis

SHAP (SHapley Additive exPlanations) analysis derives from game-theoretic Shapley values, fundamentally allocating the difference between predicted and baseline values equitably among input features. This is achieved by computing the average marginal contribution of a feature across all possible feature subset permutations, assigning each feature a SHAP value per prediction instance.

SHAP delivers highly interpretable local feature importance while enabling global importance assessment through value aggregation. Its distinctive strengths include theoretical rigor grounded in cooperative game theory; methodological unification of interpretability approaches; interaction detection between features; and visual interpretability through intuitive plots that elucidate complex model decision logic. These capabilities make SHAP indispensable for model diagnostics, feature selection, outcome interpretation, and fostering trust in model reliability.

**Figure 11:**
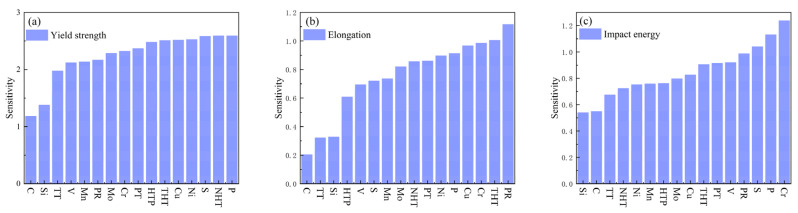
SHAP-based feature importance ranking (**a**) Yield strength; (**b**) Elongation; (**c**) Impact energy.

Figure 11a ranks feature importance for yield strength influence, revealing phosphorus (P) as the most significant factor. At low concentrations, P enhances yield strength via solid solution strengthening; however, excessive content or severe segregation induces grain boundary embrittlement, potentially causing brittle fracture. Normalizing holding time exhibits an “increase-then-decrease” effect on yield strength: insufficient duration allows progressive austenitization during extension, refining microstructure homogeneity and grain size, thereby gradually increasing yield strength. Optimal duration achieves complete austenitization with fine, uniform grains, maximizing and stabilizing yield strength. Prolonged holding coarsens austenite grains, coarsening the post-cooling microstructure and consequently reducing yield strength [28]. Sulfur (S) exhibits analogous influence to P, differing by exerting no strengthening effect at any concentration. It solely reduces strength through inclusion-induced matrix decohesion, progressively diminishing yield strength with increasing content. Conversely, Ni, Cu, Cr, and Mo enhance yield strength via solid solution strengthening and microstructural refinement (grain refinement, phase transformation optimization), serving as key alloying elements in medium-high strength steels. Tempering constitutes a critical post-quenching heat treatment, decomposing over-saturated martensite/bainite through isothermal holding, facilitating carbide precipitation and coalescence, relieving internal stresses while enabling matrix recovery and recrystallization, ultimately regulating yield strength [29].

Figure 11b ranks feature importance for elongation influence, identifying pouring speed as the dominant factor. Excessively slow pouring causes premature solidification due to rapid heat dissipation during mold filling, resulting in incomplete filling (defects) and coarse grains. Defects act as stress concentrators, initiating fracture during tensile deformation and substantially reducing elongation. Coarse grains exhibit weakened grain boundary cohesion, promoting intergranular cracking during deformation and diminishing elongation. Excessive pouring speed generates high flow velocity in the mold cavity, inducing turbulence and severe impingement that increases porosity and inclusions—disrupting metallic continuity to become fracture origins during tension, degrading elongation; simultaneously, thermal stresses and cracks directly sever matrix continuity, causing significant elongation reduction. Local grain abnormalities or segregation (e.g., uneven element distribution) further impact elongation. Optimal pouring enables stable mold filling, moderate cooling rates, defect minimization, and grain refinement, thereby enhancing elongation [30]. Tempering transforms unstable post-quench microstructures, relieves internal stresses, and modifies carbide morphology/distribution, ultimately affecting steel elongation. Insufficient tempering leaves incomplete phase transformation and residual stresses, yielding low elongation; adequate tempering achieves full martensite decomposition, carbide refinement, and stress relief, optimizing elongation; prolonged tempering causes carbide coarsening, grain growth, or surface defects, reducing elongation [29]. Alloying elements Cr, Cu, Ni, Mo indirectly influence elongation by altering crystal structure, microstructure, and solid solution strengthening effects. Phosphorus severely compromises ductility through dual mechanisms: grain boundary segregation embrittlement and dislocation motion obstruction via solid solution, dramatically reducing elongation [31].

Figure 11c ranks feature importance for impact energy influence, identifying chromium (Cr) as the predominant factor. Optimal Cr content (typically ≤5%) enhances hardenability, promoting homogeneous fine-grained microstructures (e.g., refined pearlite/bainite); concurrently, finely dispersed alloy carbides (e.g., Cr_3_C_2_) pin grain boundaries and impede crack propagation, thereby improving impact energy. However, excessive Cr (>5%) or improper heat treatment (e.g., slow cooling) promotes networked/coarse chromium carbides, causing grain boundary embrittlement where cracks readily propagate along boundaries, significantly reducing impact energy [32]. Both P and S are detrimental elements, with increasing concentrations progressively degrading impact energy. P reduces toughness, primarily through grain boundary segregation and cold brittleness, while S deteriorates toughness via brittle sulfide inclusions and hot shortness. Both excessively fast and slow pouring rates reduce impact energy due to defects (porosity, inclusions, segregation, cold shuts) or coarse structures; optimal pouring enhances impact energy by optimizing solidification structures and minimizing defects [30].Vanadium forms fine, dispersed carbonitrides (V(C,N)) with C/N, pinning grain boundaries and refining grains, benefiting toughness (grain refinement toughening). Yet coarse precipitates or excessive grain boundary precipitation may become crack initiation sites or cause localized stress concentration by impeding dislocation slip, consequently reducing impact energy.

## 4. Conclusions

1. Rigorous data preprocessing: Outliers were removed using statistical methods based on normal distribution assumptions, while oversampling techniques and normalization mitigated data imbalance and feature scale disparities, achieving significant improvements in dataset quality.
2. Multi-model performance benchmarking: Comparative analysis of BPNN, SVR, RF, and XGBoost models identified SVR as optimal, demonstrating exceptional prediction accuracy and error control.
3. Experimental validation: Newly processed specimens validated predicted tensile properties, with relative errors between measured and predicted values below 4% for all properties, confirming robust industrial applicability.
4. SHAP-based interpretability: SHAP analysis revealed dominant influence mechanisms of chemical composition (e.g., P, Cr) and process parameters (e.g., pouring speed, normalizing time) on mechanical properties, providing actionable insights for process optimization.

## Figures and Tables

**Table 1:** Data distribution after eliminating outliers.

Feature	Feature Abbreviation	Min	Max	Mean	Feature	Feature Abbreviation	Min	Max	Mean
Carbon, w%	C, w%	0.16	0.40	0.36	Pouring Rate, kg. s^−1^	PR, kg. s^−1^	192.6	678.6	372.07
Manganese, w%	Mn, w%	0.56	0.90	0.75	Holding Time after Pouring, h	HTP, h	93.0	282.0	164.6
Silicon, w%	Si, w%	0.20	0.45	0.35	Tapping Temperature, °C	TT, h	250.0	339.0	308.3
Sulfur, w%	S, w%	0.001	0.015	0.006	Normalizing Holding Time, h	NHT, h	20.0	61.0	27.0
Phosphorus, w%	P, w%	0.005	0.017	0.01	Tempering Holding Time, h	THT, h	19.0	42.5	25.8
Chromium, w%	Cr, w%	0.05	0.28	0.13	Tensile strength, MPa	UTS, MPa	524.0	630.0	560.8
Nickel, w%	Ni, w%	0.02	0.3	0.12	Yield strength, MPa	YS, MPa	250.0	350.0	295.7
Molybdenum, w%	Mo, w%	0.01	0.11	0.05	Shrinkage ratio, %	SR, %	29.0	62.0	43.7
Copper, w%	Cu, w%	0.02	0.12	0.06	Elongation, %	EL, %	21.5	30.0	26.3
Vanadium, w%	V, w%	0.001	0.007	0.003	Impact energy, J	AK, J	24.3	78.5	48.7
Pouring Temperature, °C	PT, °C	1544	1558	1551.4					

**Table 2:** Performance comparison of multiple models on the training set in terms of R^2^ and RMSE.

Properties	SVR		XGB		RF		BPNN	
	R^2^	RMSE	R^2^	RMSE	R^2^	RMSE	R^2^	RMSE
IE	0.88	5.26	0.99	0.06	0.76	5.49	0.73	5.74
TS	0.95	7.62	0.99	0.06	0.94	8.26	0.92	9.78
YS	0.98	2.86	0.99	0.05	0.93	7.09	0.92	7.67
EL	0.98	0.25	0.99	0.01	0.86	0.83	0.91	0.67
RA	0.98	0.72	0.99	0.04	0.80	2.87	0.80	2.87

**Table 3:** Performance comparison of multiple models on the testing set in terms of R^2^ and RMSE.

Properties	SVR		XGB		RF		BPNN	
	R^2^	RMSE	R^2^	RMSE	R^2^	RMSE	R^2^	RMSE
IE	0.85	5.51	0.97	1.85	0.73	5.92	0.67	6.47
TS	0.93	7.52	0.83	13.12	0.90	10.12	0.89	10.48
YS	0.95	7.59	0.97	4.85	0.93	8.33	0.82	13.56
EL	0.89	0.68	0.90	0.64	0.81	0.89	0.84	0.81
RA	0.95	1.47	0.93	1.75	0.67	3.96	0.64	4.14

**Table 4:** Sample workpiece chemical composition and process parameters.

Feature	Sample 1	Sample 2	Sample 3	Sample 4	Feature	Sample 1	Sample 2	Sample 3	Sample 4
C, w%	0.39	0.37	0.37	0.37	PR, kg. s^−1^	428.96	417.55	393.26	208.00
Mn, w%	0.72	0.82	0.73	0.81	HTP, h	160	183	158	144
Si, w%	0.32	0.35	0.32	0.35	TT, h	308	313	323	250
S, w%	0.002	0.002	0.003	0.008	NHT, h	23.0	23.5	23.0	28.0
P, w%	0.014	0.014	0.015	0.009	THT, h	27.0	27.0	27.5	23.0
Cr, w%	0.17	0.22	0.17	0.11	TS, MPa	558	555	552	559
Ni, w%	0.12	0.13	0.13	0.20	YS, MPa	292	304	287	293
Mo, w%	0.05	0.04	0.03	0.07	RA, %	47	44	43	43
Cu, w%	0.07	0.06	0.06	0.05	Elongation, %	26.0	26.0	26.5	28.0
V, w%	0.005	0.006	0.005	0.002	IE, J	53.57	53.40	48.47	45.77
PT, °C	1549	1553	1551	1558					

## Data Availability

The original contributions presented in this study are included in the article. Further inquiries can be directed to the corresponding authors.
